# Salmagundi

**Published:** 1885-09

**Authors:** 


### SALMAGUNDI.

EDITED BY E. G. BETTY, D. D. S.

THERE is a dentist in Philadelphia who is going to make a fortune. He has discovered that the rinsing of the mouth with brandy is better than a brush for cleansing the teeth.—*Clipping.*

EDITOR SALMAGUNDI.

IN the August "Century" Howells describes an old Italian woman " with a single tooth rising like an obelisk from her lower jaw"! He might have added that this stood as a monument to the glory of the departed grinders!—K. C. M.

"ANOTHER new metal, is now added to our rapidly growing list of elements. It was discovered by Dr. T. Dahll, in examining a specimen of nickel ore from Kragero, in Norway. It is a malleable metal, of white color, with a tinge of brown; it presents, when pure, a metallic luster, but, on exposure to atmosphere, becomes coated with a thin film of oxide; its hardness is about that of copper, and its specific gravity is 9.4441. At 350 degrees C. it melts. From its physical properties and chemical reaction it appears to differ from every other known metal, and Dr. Dahll claims for it a distinct individuality."

The name of the above new metal we failed to get, may be some of our readers know it and will be good enough to tell us.

---

BUCYRUS, O., August, 10th, 1885.

EDITOR SALMAGUNDI.

I would like to ask Dr. Williams, to explain the last two lines of his article upon "Crown Work" in August Register. He says: "One other advantage in case of accident to the porcelain, is the great ease with which another crown can be attached."

Now if the post is securely locked in position and the "key thrown into the well" how is the post *easily* to be soldered to another crown?

We all know it is no very easy task to remove a properly set post from a root. I may be dull of comprehension, but see no other way. Will the Doctor explain? D. S. DIBBLE.

---

TEETH IN THE NOSE.—A singular case is reported by Dr. Branch of a small colored boy in Basseterre, West Indies, who has two large teeth in his nose. A portion of his nose having been destroyed by ulceration, he was put under medical treatment for that disease, which soon healed with the application of chloroform ointment and the administration of iodide of potassium. The Doctor's intention was to have made a new nostril for the boy, but while awaiting to make sure of the cure of the ulcer, these teeth began to make their appearance. Dr. Branch says that they seem to be the two central incisors of the permanent set; they spring from the floor of the nostrils at the sides of the bony septum, are freely movable, and are apparently attached to the mucous membrane only; they have little or no roots, but large crowns.—*New York Tribune.*

---

CHEAPEST DISINFECTANT KNOWN.—A chemist in Chicago wrote to the *Inter Ocean*, of that city, the following communication on the above subject which is worthy of reproduction in the "REGISTER."

Take one-eighth of an ounce of nitrate of lead, and dissolve in one quart of boiling water, then dissolve one ounce of common salt in five gallons of water. Pour the two solutions together, and when settled pour the clear solution from the sediment, and keep well corked in a demijohn or jug for use.

A cloth wet with this and suspended in the room will neutralize all offensive vapors, and a little dashed into a privy, sink, drain, or sewer will disinfect and destroy all noxious gases by combining with them. It is said to be in general use in England for purifying sewers, also for destroying bilge water in the holds of vessels.

The above from a London correspondent, was taken from the New York Herald in 1879. As the result of the mixture is a solution of chloride of lead, I concluded to give it a trial, at the same time fearing the solution would be too attenuated to be effective. It proved, however, to be all that is claimed for it, and I have used it during the last five years with entire satisfaction. The materials from which it is made costs less than one-half cent a gallon, and the manipulation is simple.

I believe that a trifling expenditure would deodorize our sewers and relieve us from the nauseating stench that rises through the ventilators in the streets.

---

VINCENNES, IND., August 16.—The developments concerning the new medical State law have become very surprising to not a few physicians. The stringency of the law is what surprises and astonishes a large number of the medical fraternity. If a practicing physician fails to obtain a license from the County Clerk he subjects himself to a fine of from $10 to $200, and if a physician waits on a patient now without having first obtained a license, that patient need not pay the physician for his services, and the physician has no right under the law to charge for his services.

A physician may obtain a license on the following conditions: Applicant must show by his affidavit that he is a regular graduate and show his diploma to the Clerk; or, applicant must show by affidavits of himself and of two freeholders that he or she has resided in the State and practiced medicine, surgery and obstetrics in the State continuously for ten years; or, when applicant shall file affidavit with the Clerk to the effect that he has resided in the State for three years and attended one full course of lectures in some reputable college. When the physician has done either of the above he may receive a license from the Clerk. A physician moving from one county to the other must also take out a new license, as the license in the county where he formerly lived is void after removal. As a general thing physicians pay but little attention to the law, and not one-tenth of them will secure a license until they are compelled."

The above clipping from a daily paper, notices a law the like of which would benefit dentistry very much and we guarantee that more attention would be paid to it by dentists than the physicians.

---

How artificial teeth may do damage.—Another agent in the combination to maintain for the man of advancing age his career of flesh-eater is the dentist. Nothing is more common at this period of life than to hear complaints of indigestion experienced, so it is affirmed, because mastication is imperfectly performed for want of teeth. The dentist deftly repairs the defective implements, and the important function of chewing the food can henceforth be performed with comfort. But, without any intention to justify a doctrine of final causes, I would point out the significant fact that the disappearance of the masticating powers is mostly coincident with the period of life when that species of food which most requires their action—viz., solid animal fiber—is little, if at all, required by the individual. It is during the latter third of his career that the softer and lighter foods, such as well-cooked cereals, some light mixed animal and vegetable soups, and also fish, for which teeth are barely necessary, are particularly valuable and appropriate. And the man with imperfect teeth who conforms to nature's demand for a mild non-stimulating dietary in advanced years will mostly be blessed with a better digestion and sounder health than the man who, thanks to his artificial machinery, can eat and does eat as much flesh in quantity and variety as he did in the days of his youth. Far be it from me to undervalue the truly artistic achievements of a clever and experienced dental surgeon, or the comfort which he affords. By all means let us have recourse to his aid when our natural teeth fail, for the purpose of vocal articulation, to say nothing of their relation to personal appearance; on such grounds the artificial substitutes rank among the necessaries of life in a civilized community. Only let it be understood that the chief end of teeth, so far as mastication is concerned, has in advancing age been to a great extent accomplished, and that they are now mainly useful for the purposes just named. But I cannot help adding that there are some grounds for the belief that those who have throughout life from their earliest years consumed little or no flesh, but have lived on a diet chiefly or wholly vegetarian, will be found to have preserved their teeth longer than those who have always made flesh a prominent part of their daily food.—*Popular Science Monthly.*

---

CINCINNATI, August 11, 1885.

EDITOR SALMAGUNDI:

The wisest of all men once upon a time, made this broad assertion: "There is nothing new under the sun." I am lead to believe that the old scientist had just been suspended from the "Society of Natural History," in Jerusalem, for the nonpayment of his dues, when he promulgated the above.

Neither time nor space will permit me at present to convince the readers of "SAL." that *Sol* was a little "off" on this point at least.

I will report something *new*, at least to me, which came under my notice some two months ago.

Some seven years ago, an elderly gentleman called upon me for professional services. Upon examination of the mouth I found the upper centrals and laterals (but am not certain about the canines) free from decay, but very loose from accumulations of tartar; the whole mouth presented a soft and flabby condition. I extracted those teeth, also loose teeth and roots in the inferior maxilla. In due time I substituted a full upper and lower denture; everything went along smoothly until about a year ago he complained of his upper plate becoming very loose, owing to two roots, as he thought, working their way through the gum.

Two months ago he called on me to have those roots extracted, when to my utter astonishment, I found upon examination the two supposed roots to be full grown, well developed and healthy right and left canines, lying at an angle of about 45 degrees, the points making strenuous efforts to meet at the median line; they seem perfect in shape and formation, and are very large; the mouth at present presents a healthy condition with a complete absorption of the processes. He is sixty years of age, has been and is at present enjoying good health.

Now the question which naturally arises, is it second or third dentition?

Dr. Chapin Harris and others report cases of full upper and lower teeth, third dentition; others claim that such an anomaly is in direct conflict with physiological and natural laws.

As my memory of the presence or absence of the canine teeth when I extracted those adjoining them is not clear, this case must rest in doubt.

I would impress upon the minds of my young brothers, as well as on my own, the great importance of bein gmore observing in our practice; duly recording each and every operation, trivial or important, then at any future time we can refer to the history of each case and come to a definite conclusion and establish facts regarding so-called dental anomalies of to-day.

I also have a patient sixty-five years of age, who is cultivating an anomaly which promises to develop into a right superior canine, in the near future. Until then, progress,

Yours cohesively, W. D. PHILLIPS.

